# Influence of Surface Modification on the Microstructure and Thermo-Mechanical Properties of Bamboo Fibers

**DOI:** 10.3390/ma8105327

**Published:** 2015-09-24

**Authors:** Xiaoping Zhang, Fang Wang, Leon M. Keer

**Affiliations:** 1 Faculty of Materials and Energy, Southwest University, Chongqing 400715, China; zhangxiaopin@swu.edu.cn; 2 Department of Mechanical Engineering, Northwestern University, 2145 Sheridan Road, Evanston, IL 60208, USA; l-keer@northwestern.edu

**Keywords:** natural fiber, alkali treatment, thermal properties, mechanical performance, testing

## Abstract

The objective of this study is to investigate the effect of surface treatment on the morphology and thermo-mechanical properties of bamboo fibers. The fibers are subjected to an alkali treatment using 4 wt % sodium hydroxide (NaOH) for 1 h. Mechanical measurements show that the present concentration has an insignificant effect on the fiber tensile strength. In addition, systematic experimental results characterizing the morphological aspects and thermal properties of the bamboo fibers are analyzed by scanning electron microscopy, Fourier transform infrared spectroscopy, thermogravimetric analysis, and differential scanning calorimetry. It is found that an alkali treatment may increase the effective surface area, which is in turn available for superior bonding with the matrix. Fourier transform infrared spectroscopy analysis reveals that the alkali treatment leads to a gradual removal of binding materials, such as hemicellulose and lignin from the bamboo fiber. A comparison of the curve of thermogravimetric analysis and differential scanning calorimetry for the treated and untreated samples is presented to demonstrate that the presence of treatment contributes to a better thermal stability for bamboo fibers.

## 1. Introduction

In recent years, there has been increasing attention given to the use of biodegradable composites reinforced with natural fibers with a view towards economic incentives and environmental concerns [1,2]. Natural fibers are widely accepted to be a potential replacement as reinforcement for use in petroleum-based polymers. The advantages of plant-based fibers over traditional synthetic fillers are their light weight, low cost, specific mechanical properties and excellent biodegradable properties. There is evidence that shows that the presence of natural fiber in polymeric materials can enhance the performance of virgin materials [3]. Among the many different types of such materials, bamboo fiber itself is a unidirectional reinforcing composite consisting of long and parallel cellulose fibers embedded in an amorphous matrix of lignin and hemicellulose [4], and then is recognized as one of the most attractive reinforcement fillers because of its excellent inherent properties [5,6]. In addition, the inexhaustible supply and natural abundance of bamboo make it an excellent candidate for developing natural composites. Thus, over the last two decades, the use of bamboo fibers as a reinforcing material in structural composites has gained popularity in the building and automotive industries [7,8].

There is no denying that the properties of a biocomposite are mainly influenced by the interaction between hydrophilic natural fibers and a hydrophobic matrix [9]. Compared to traditional inorganic fibers such as glass and carbon, the major disadvantages of natural fibers are their high moisture absorption and weak interfacial bonding to the thermoplastic matrix [10,11] and that is the reason that these fibers have not completely replaced conventional fiber materials in high-load applications. Recently, Ngo *et al.* performed an in-depth study on the compostability and mechanical properties of thermoset composites reinforced with natural fibers [12,13,14]. The results show that an addition of a tertiary oil phase can increase the material ductility and leads to the better adhesion between the fibers and the polymer matrix. It is well known that the major constituents of natural fiber consist of cellulose, hemicellulose, and lignin. The other extra components, such as pectin and waxy substances, are usually regarded as surface impurities [15]. To make natural fibers a suitable reinforcing agent with adequate bonding characteristics for general application, various modification methods, including alkali treatment, graft modification, and infiltration, are used to improve interfacial compatibility. Alkali treatment is one of the effective modification methods and is a way to increase the interfacial adhesion by removing hemicellulose, lignin, and other components from natural fiber, resulting in better-purified cellulose [16]. Some reports claim that the surface area of the alkali-treated abaca fibers is increased in comparison to untreated fibers, which in turn leads to better interfacial adhesion of the fiber with its surrounding matrix [17]. Natural fibers such as jute, hemp, and kenaf have been investigated for surface modification [18,19,20]. Although some work has been devoted to investigate the mechanical behavior of alkali-treated bamboo fiber reinforced composites [21,22], a comprehensive study is still required to provide an understanding of the microstructure and thermo-mechanical properties of bamboo fibers before and after alkali treatment.

The difficulties in characterizing the properties of bamboo fibers are increased as a result of the complexity of their overall microstructure, which by far exceeds that of synthetic materials and other natural fibers. This complexity is caused by the presence of different materials in variable proportions such as cellulose, hemicellulose, lignin, and pectin [11]. A few studies have been performed on the thermo-mechanical behavior of bamboo fibers, but little knowledge of interfacial bonding has been obtained. The present work attempts to perform a systematic investigation of the influence of alkali treatment on bamboo fibers through multiple analytical techniques from microscopic to macroscopic level. Scanning electron microscopy (SEM), Fourier-transform infrared spectroscope (FTIR), thermal gravimetric analysis (TGA), and differential scanning calorimeter (DSC) are used to analyze the changes in morphology, chemical composition, and thermal characteristics occurring in bamboo fibers due to treatment with a concentration of NaOH solution. The effect of chemical treatment on the mechanical properties of bamboo fibers is also examined. The structural and chemical information obtained, combined with thermo-mechanical properties on the untreated and treated fibers, serves as a guide to the development of surface modification of bamboo fibers for advanced composite applications.

## 2. Experimental Section

### 2.1. Fiber Preparation

The bamboo tested in the present work was selected from one of the most popular bamboo species in China, known as Moso bamboo and is supplied by the Jian Zhou Group (Fujian, China). The manually decorticated bamboo strip specimens were predried in a vacuum oven at 105 °C to vaporize the water, and then cut into a series of sheets of a fiber diameter between 120 and 180 μm. Because the fiber strips removed from the culms contained tissues and gums, the dry fibers were extracted by means of a chemical process of decomposition called degumming by enzymes fermentation, in which the gummy materials were eliminated. After these steps, the rough machined fibers were further combed and cooked, first with warm water for 1 h (90 °C), then wiped with ethanol with a piece of cotton tissue. Finally, a batch of samples with length and diameter of about 8 cm and around 153 μm were obtained for measurements.

### 2.2. Alkali Treatment

The bamboo fibers from the previous preparation were soaked in a NaOH solution (4% by weight) at room temperature, maintaining a liquor ratio of 20:1. The fibers were immersed in the alkali solution for 1 h. Afterwards, the fibers were continuously washed with distilled water, removing any NaOH solution remaining on the fiber surface; neutralized with dilute acetic (3% by weight); and then washed again with water. The neutrality of pH = 7 was checked with litmus paper. Finally, the fibers were dried at room temperature until a constant weight was reached. The dried fibers were stored in a desciccator with a sealed plastic bag to avoid atmospheric moisture contamination prior to chemical and thermo-mechanical analyses. The nomenclature for the fibers used in this work is as follows: the abbreviation “UTBF” denotes untreated bamboo fibers, and “ATBF” denotes alkali-treated bamboo fibers.

### 2.3. Measurements

#### 2.3.1. Morphological Characterization

Morphological studies of treated and untreated bamboo fibers were conducted by SEM, using a JMS-6610 machine (JEOL, Tokyo, Japan). The samples were all sputter-coated with gold to a thickness of ~10 nm before surface examination in order to prevent the accumulation of electrostatic charge under the electron beam. The SEM was equipped with a tungsten filament as an electron emitter source. Accelerating voltages were used to collect the SEM images before and after treatment.

#### 2.3.2. FTIR Spectroscopy

In order to analyze the conformational changes that occurred, FTIR spectra of UTBF and ATBF were recorded on a Bruker, TENSOR27 FTIR spectrometer (Ettlingen, Germany) for infrared representation in the wavenumber range of 600–3900 cm^−1^ with a resolution of 0.01 cm^−1^. An average of 10 scans was recorded for each spectrum. The specimen was crushed into small particles in liquid nitrogen so that a point-to-point contact could be conducted with the pressure device.

#### 2.3.3. TG and DSC Analysis

TG-DSC simultaneous thermal measurement (Netzsch STA409C, Selb, Germany) was adopted to conduct the thermal analysis of UTBF and ATBF. A sample of about 15 mg was weighed and sealed for the TG-DSC analysis. All tests were performed in the temperature range from 25 to 600 °C under a nitrogen atmosphere. A constant heating rate of 10 °C/min was maintained.

#### 2.3.4. Mechanical Testing

**Figure 1:**
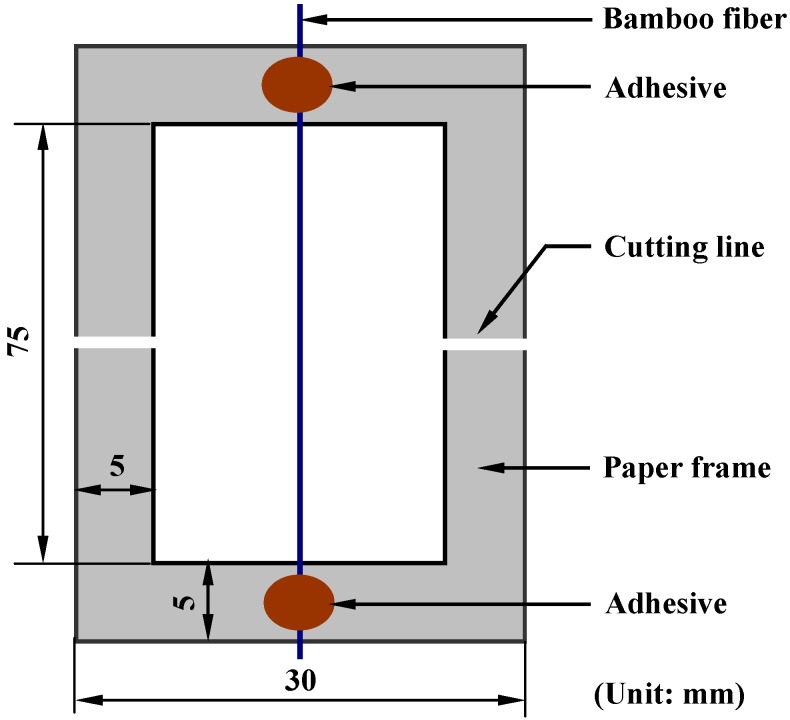
Use of a paper frame to glue fiber specimen.

The tensile strengths of bamboo fibers were measured in accordance with the preparation procedure described in ASTM D3822-07 [23]. Mechanical tests were conducted on a universal testing machine (Model 3369, Instron Corporation, Grove, PA, USA) and performed at ambient conditions with a cross head speed of 0.5 mm/min in a displacement control mode. Each fiber specimen was glued onto a stiff paper frame with a gauge length of 7.5 cm. To position the fibers as straight as possible between the clamps, the ends of the fibers were glued with a double-sided adhesive onto the paper frame, as shown in Figure 1. Upon clamping the ends of the supporting frame by the jaws of the testing machine, the frame edges were carefully cut in the center.

The test was carried out for 30 specimens to get a valid average. Tensile strength was calculated from the load-elongation data and cross-sectional area of fibers. The cross-sectional area, assuming that the fibers are cylindrical in shape, was evaluated from the diameter measured using an optical microscope at five different locations along each sample length. It is noted that results should be discarded when the fracture occurred at the clamp or when grip sliding occurred during the stretching process.

## 3. Results and Discussion

### 3.1. Structural Characteristics

**Figure 2:**
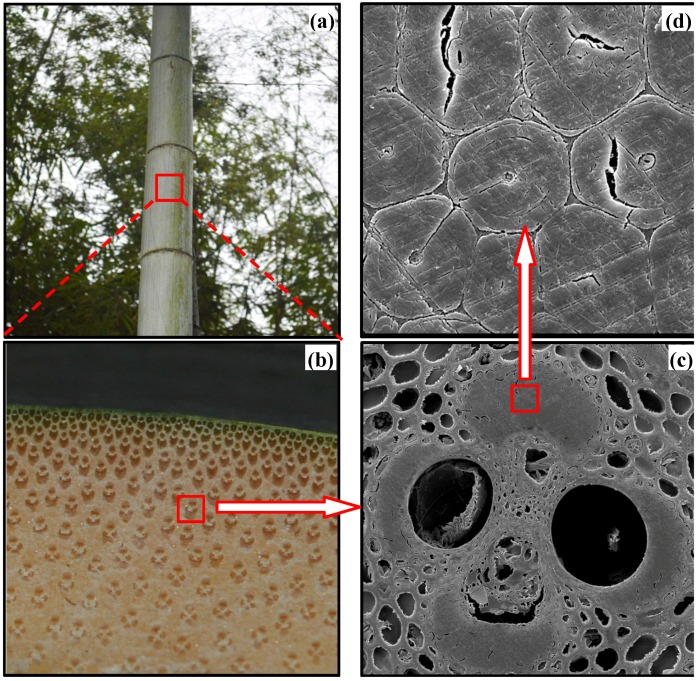
Hierarchical structure of bamboo fiber across different length scales. (**a**) Bamboo culm; (**b**) Cross section of the culm; (**c**) Vascular bundle; (**d**) Elementary fibers.

Figure 2 shows a group of bamboo structures from the macroscopic to microscopic level. The often-called technical fibers are extracted from the bamboo culms using an extraction process as in [24]. The basic tissues comprising all herbaceous plants are epidermal, vascular and hypodermis [25]. Vascular bundles are commonly known as bamboo fibers [5]. As can be seen in Figure 2b, the content of vascular bundles tends to a gradient variation from the outer to inner layer of a piece of bamboo. At the outer periphery, vascular bundles are compacted and are more numerous than the bundles at the inner zone. On the other hand, the occupied volume of the sparsely distributed vascular bundles is larger in the inner layer. Such a gradient structure is commonly observed in biological plants. Recent research indicates that the graded surface can provide an appealing prospect for controlling surface damage and failure during repeated contact, leading to a material performance improvement [26]. Figure 2d shows a SEM micrograph of the microstructure of a vascular bundle, indicating that a bamboo fiber consists of considerable elementary fibers [6].

### 3.2. Surface Profiles

Analogous to other lignocellulosic reinforcing plants, bamboo is a multicellular composite fiber. Each unit cell of bamboo fiber is composed of cellulose microfibrils with different fibrillar orientations surrounded and cemented together with lignin and hemicellulose [27]. These cells extend longitudinally overlapping each other. Because of this overlapping nature, a mesh-like structure arises. The NaOH reacts with hydroxyl groups of the cementing material hemicellulose and brings on the destruction of the cellular structure and thereby the hydrophilic nature of the fiber degrades [28]. The following reaction will occur under the action of alkali on the surface of bamboo fiber:

(1) Bamboo–OH + NaOH ⇄ Bamboo–O^−^ Na^+^ + H_2_O

**Figure 3:**
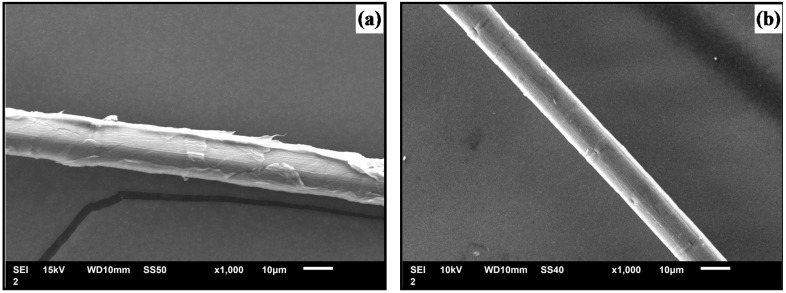
SEM images of (**a**) untreated bamboo fiber and (**b**) alkali-treated bamboo fiber.

Typical SEM images of the lateral section of UTBF and ATBF are shown in Figure 3, which display the surface morphology of the bamboo fibers before and after treatment. It can be seen that many uplifted filaments are packed together on the surface, as indicated in Figure 3a. Okubo *et al.* reported that the uplifted materials are organic [29]. Owing to the weak binding, they can be easily separated from the fiber surface by external force. After treatment, these filaments disappear and the fiber surface becomes clean and is attached with some joints and tiny fissures, which can be attributed to the elimination of wax, hemicellulose, and other inorganic impurities, as seen from Figure 3b. This could result in an effective fiber surface increment available for interlocking adhesion with the matrix resin as the morphology changes. Moreover, splitting of the cemented fibers causes a reduction in the fiber diameter, leading to a higher aspect ratio. It is concluded from the results that when the fibers are used as reinforcing agents, alkali treatment plays an important role in enhancing the interfacial bonding.

### 3.3. Infrared Spectroscopic Analysis

**Figure 4:**
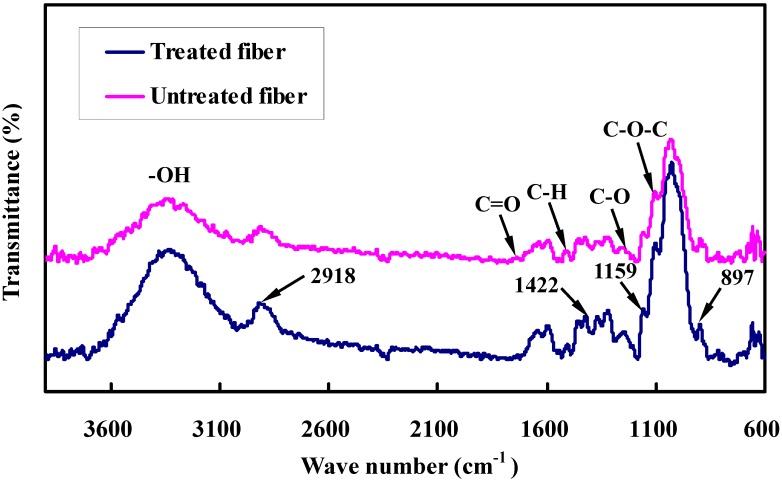
FTIR spectra of untreated and alkali treated fibers.

FTIR can help identify the functional groups present in the fiber, thereby highlighting the chemical differences among the fiber constituents. Figure 4 represents the FTIR spectra of untreated and alkali-treated bamboo fibers. The exact position and potential assignments of the peaks are listed in Table 1.

**Table 1:** Infrared transmittance peaks of fiber constituents.

Wavenumber (cm^−1^)	Possible Assignments	Reference
3200–3500	–OH stretching vibration in cellulose, hemicellulose and lignin	[30]
2918	C–H stretching vibration in methyl and methylene	[19]
1737	C=O stretching vibration of hemicellulose and pectin	[17,18]
1513	C–H stretching vibration of hemicellulose and pectin	[17]
1422, 1159	Typical absorption peak of cellulose	[31]
1247	Acetyl groups of lignin	[32]
1104	C–O–C stretching vibration of cellulose	[17]
1032	C–O/C–C stretching vibration	[33]
897	C–OH stretching vibration, which indicates β-glycosidic linkage between the monosaccharides	[34]

Due to OH stretching vibration, the peak area within the region 3200–3500 cm^−1^ is found to be intensified after NaOH treatment and indicates that some alkali-sensitive material is wiped off during alkali treatment, resulting in the rising percent of hydroxyl groups. Specifically, the peak located in 2918 cm^−1^ is a hydrocarbon (C–H) stretching vibration. In addition, the peaks observed at about 1422 cm^−1^ and 1159 cm^−1^ are the typical absorption peak of cellulose. Compared to the untreated fibers, these absorption peaks appear distinctly as a result of treatment. It is emphasized that the peak is shifted to the lower value of 1032 cm^−1^ derived from C–O and C–C stretching vibration, which may be expected as a result of the change in molecular orientation by alkaline solution.

The characteristic peaks located at about 1737, 1513, 1247, 1104, and 897 cm^−1^ are designated as the peak of C=O, C–H, C–O, C–O–C, and C–OH, respectively. The peak at 1737 cm^−1^, which is related to the C=O bond of the carboxylic group of hemicelluloses and pectin, is seen in the untreated samples but becomes weaker with the alkali treatment. The result could be attributed to the fact that these constituents cannot be completely removed in the dilute alkali solution. The peak 1247 cm^−1^ that is assigned to the C–O stretching vibration of acetyl groups of lignin is well defined in the untreated fibers. These phenomena are an indication of the removal of hemicellulose, pectin, and part of lignin from bamboo fibers during alkali treatment. A comparison of the FTIR spectra of UTBF with ATBF shows that the locations of the peak positions of these samples have no significant deviation but an obvious difference in peak intensity, indicating that hemicellulose and impurities on the fiber surfaces are partly removed by the NaOH treatment although no new groups are introduced in the cellulose molecules.

### 3.4. Thermal Property Analysis

Fiber constituents degrade at different temperatures and the result is weight loss [19]; TGA is then performed to examine the thermal stability of bamboo fiber. The percent weight loss of the sample was calculated as

**Figure 5:**
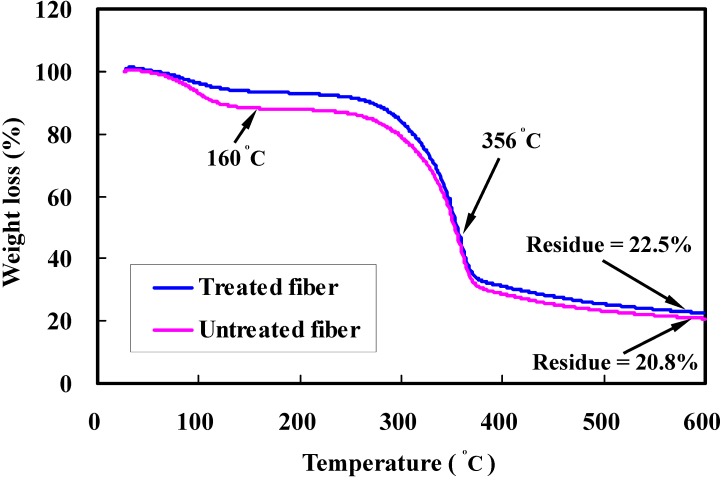
TGA curve of untreated and alkali treated bamboo fibers.

(1) Percent Weight loss = (*W*_1_ − *W*_2_)/*W*_1_ × 100%

where *W*_2_ and *W*_1_ correspond to the sample weight at any given temperature and prior to testing. The effect of rising temperature on the mass change of untreated and alkali-treated bamboo fibers is illustrated in Figure 5. 

It can be visually observed that the alkalized fibers have less weight loss than the untreated specimen. The degradation profiles of the untreated fibers are distinctly characterized by three weight loss steps. Weight loss in the first step occurs below 100 °C due to moisture evaporation from the fiber structure. The corresponding mass loss is approximately 10%. Compared with the UTBF, NaOH treatment would decrease the hydrophilicity of the bamboo fibers. In the following thermal decomposition step, the temperatures are concentrated in the range of 160 to 356 °C, and the weight loss is mainly caused by the decomposition of hemicellulose and cellulose. Previous studies on hemp fibers reveal that cellulose is entirely decomposed under a higher temperature range (250 to 350 °C) than that of hemicellulose (180 to 280 °C) [19]; thus the decomposition process occurs mainly on the cellulose, which in turn increases the overall degradation temperature of the treated fibers, leading to higher thermal stability. The third step ranging from 356 to 450 °C is derived from decomposition of lignin. Since lignin is more difficult to decompose, it has a lower decomposition rate and is degraded in a broad temperature range. The difference in mass loss between the untreated and treated fibers gives further evidence of hemicellulose and lignin constituents dissolved and removed from the fiber during the alkali treatment. When the temperature is above 450 °C, the fibers are thoroughly degraded so that the residual mass remained unchanged with increasing temperature. The ash which remained at the end of the TG analysis for the sample is about 20% of the total sample weight. It can be seen that the alkali treatment leads to a gradual removal of non-cellulosic materials, such as hemicellulose and lignin, from the raw fibers, and indicates that highly pure cellulose in natural fibers could enhance their thermal stability [35].

**Figure 6:**
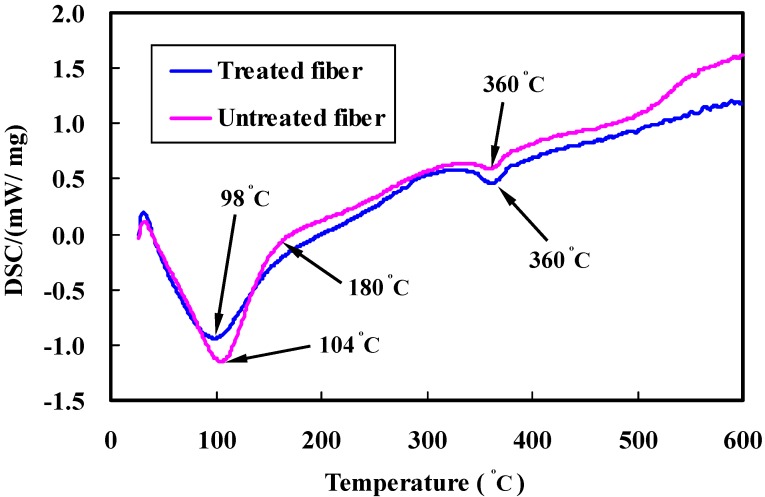
DSC analysis of untreated and alkali treated bamboo fibers.

DSC allows a measurement of chemical changes among the fiber constituents when subjected to heat. Figure 6 shows the DSC curves for untreated and treated fibers.

In the case of UTBF, the endothermic peaks at around 100 °C are strengthened, the cause of which is the evaporation of free water in the samples. However, the enthalpy change value is lower and the endothermic peak shifts to a lower temperature compared to the untreated fibers, which is from 104 to 98 °C. This phenomenon could be explained by noting that an increase in the length diameter ratio of fiber during pre-treatment with NaOH helps facilitate evaporation of moisture at a lower temperature. As compared in the figure, the DSC curve shows an obvious exothermic peak at around 180 °C in the untreated fiber, which is derived mainly from the degradation of hemicellulose. This can be attributed to partial removal of the hemicellulose in bamboo fibers by alkali treatment. Another endothermic peak at around 360 °C is attributed to the degradation of cellulose. The phenomenon that the peak is weakened in untreated fibers results from the alkali treatment leading to a gradual removal of binding materials, such as hemicellulose and lignin, from the raw fibers, which increases the relative amount of cellulose contents [15].

### 3.5. Tensile Strength

**Figure 7:**
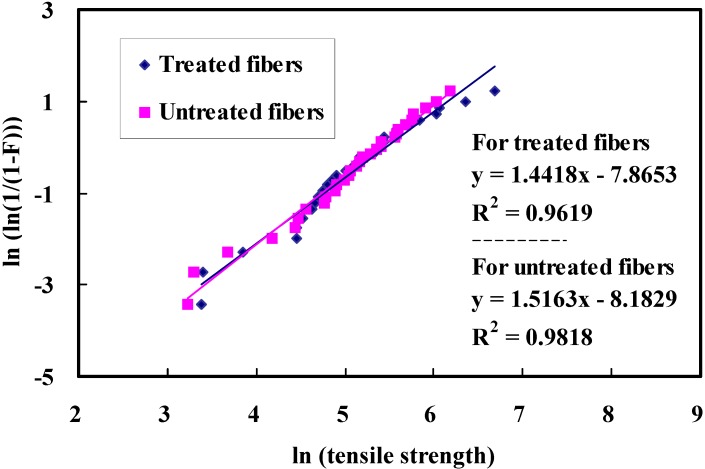
Weibull plots of the tensile strength of bamboo fibers before and after alkali treatment.

Weibull plots of the fracture strength for treated and untreated bamboo fibers are shown in Figure 7. Here, *F* is the cumulative failure probability of the fiber [36]. The distributions of colored dots are the experimental results, and the lines represent the straight line fit for those data points. In all cases, the *R*^2^ coefficient is relatively high (over 0.96), which indicates a good degree of linearity. Thus, the fracture strength of bamboo fibers can be described effectively by the Weibull function.

**Figure 8:**
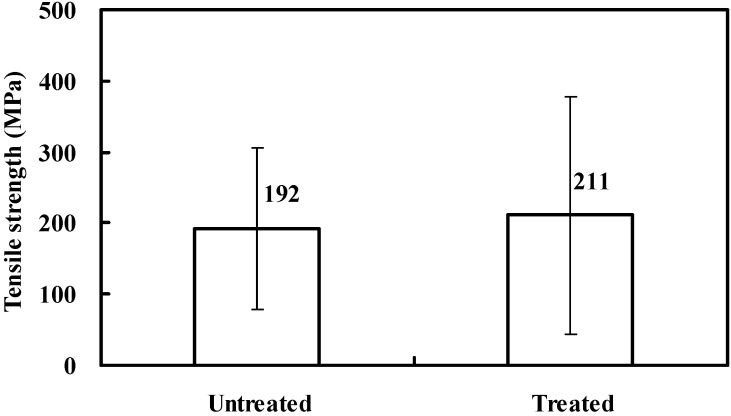
Tensile strength of bamboo fibers before and after alkali treatment.

Figure 8 shows the fracture strength of untreated and treated bamboo fibers. For each data point, 30 samples are tested and the value given is the mean of those values. The standard deviation obtained is included in the figure. The fracture strength of the fibers after treatment is 211 MPa in contrast to 192 MPa. An improvement of about 10% in average strength is achieved by alkali treatment, which may be attributed to an increment in the packing density of cellulose molecular and effective fiber surface area available for interaction with the matrix after removing the cementing materials. However, it can be seen from the standard deviations that the strength variability is insignificant for these data sets. There are two potential reasons. One is derived from variable geometrical structure of bamboo fibers. Since bamboo fibers always exhibit between-fiber and within-fiber diameter variations, there are random fluctuations in fiber strengths. The second is derived from dilute alkali solution. In untreated bamboo fibers, hemicellulose remains dispersed in the interfibrillar region separating the cellulose chains from one another. The cellulose chains are therefore always in a state of constraint [37]. However, only part of the inorganic impurities can be removed by the low alkali concentration, thus the internal constraint cannot be totally removed and the fibrils are incapable of rearranging themselves in a compact manner along the direction of tension force [27]. As a consequence, such a concentration has a minor effect on the tensile strength of bamboo fibers. It is emphasized that the influence of alkali concentrations on the mechanical properties of bamboo fibers needs to be accounted for in further study. 

## 4. Conclusions

This study aims to use its findings as a basis for exploiting new natural reinforcements for designing superior green composites. In the present work, the effects of alkali treatment on the internal microstructure and thermo-mechanical properties of bamboo fibers are systematically analyzed by multiple measurements. The results obtained would be useful for the understanding of properties of bamboo fiber and its composites. They are summarized as follows:

1. Morphological changes by micrograph observations show that an alkali treatment leads to an increase in fiber surface area available for interlocking adhesion with the matrix resin, resulting in superior interfacial bonding over untreated ones.
2. FTIR analysis confirms that removal of hemicellulose and lignin can increase the relative amount of cellulose content in the treated fibers. Furthermore, no new groups are introduced in the cellulose molecules after alkali treatment, as evidenced by spectra measurements.
3. TGA-DSC testing reveals that surface treatment can influence chemical structure of bamboo fibers. Treated fibers exhibit higher thermal stability compared to untreated fibers, since the binding materials such as hemicellulose, pectin, and lignin can be diminished from the bamboo fibers by alkali reaction. Experimental results also suggest that hemicellulose is the most reactive constituent and is more easily degraded than the cellulose and lignin. Cellulose exhibits better thermal stability and lignin is degraded in a wide range of temperatures.
4. The average fracture strength of treated fiber on the condition of 4 wt % NaOH for 1 h is increased by 10% compared to the untreated fibers. Alkali treatment can reduce the hydrophilicity of bamboo fiber, which might in turn improve the interfacial bonding. However, it is found from the standard deviations that the present concentration has a minor effect on the tensile strength of bamboo fibers.
